# Adapting a generic tuberculosis control operational guideline and scaling it up in China: a qualitative case study

**DOI:** 10.1186/1471-2458-8-260

**Published:** 2008-07-29

**Authors:** Xiaolin Wei, John D Walley, Xinyuan Liang, Feiying Liu, Xiulei Zhang, Renzhong Li

**Affiliations:** 1 Nuffield Centre for International Health and Development, University of Leeds, UK; 2 Guangxi Centre for Disease Control and Prevention, PR China; 3 Shandong Centre for TB Control, PR China; 4 China National Centre for TB Control and Prevention, PR China

## Abstract

**Background:**

The TB operational guideline (the *deskguide*) is a detailed action guide for county TB doctors aiming to improve the quality of DOTS, while the China national TB policy guide is a guide to TB control that is comprehensive but lacks operational usability for frontline TB doctors. This study reports the process of deskguide adaptation, its scale-up and lessons learnt for policy implications.

**Methods:**

The deskguide was translated, reviewed, and revised in a working group process. Details of the eight adaptation steps are reported here. An operational study was embedded in the adaptation process. Two comparable prefectures were chosen as pilot and control sites in each of two participating provinces. In the pilot sites, the deskguide was used with the national policy guide in routine in-service training and supervisory trips; while in the control sites, only the national policy guide was used. In-depth interviews and focus groups were conducted with 16 county TB doctors, 16 township doctors, 17 village doctors, 63 TB patients and 57 patient family members. Following piloting, the deskguide was incorporated into the national TB guidelines for county TB dispensary use.

**Results:**

Qualitative research identified that the deskguide was useful in the daily practice of county TB doctors. Patients in the pilot sites had a better knowledge of TB and better treatment support compared with those in the control sites.

**Conclusion:**

The adaptation process highlighted a number of general strategies to adapt generic guidelines into country specific ones: 1) local policy-makers and practitioners should have a leading role; 2) a systematic working process should be employed with capable focal persons; and 3) the guideline should be embedded within the current programmes so it is sustainable and replicable for further scale-up.

## Background

Guideline adaptation is an important tool for knowledge transfer in public health interventions. International organisations for developing countries, such as the World Health Organisation (WHO), have developed a number of generic case management guidelines such as the WHO tuberculosis (TB) treatment guideline [1,2]. The usual emphases are to make these guidelines evidence-based with concise instructions [3]. The guidelines are then introduced to developing countries, typically with little changed to reflect the country-specific policy and settings; these changes are implicitly assumed to be the national programme's responsibility but are often not made [4]. As a result, international guidelines are often not used in practice. In the adaptation process, various issues from patients and providers have to be considered e.g., barriers to health systems access, specific national policies, the social complexity of human behaviour change, competing development goals faced by policy-makers, and resource constraints [5-7]. Usability features, such as the acceptability, comprehensibility and practicability of guidelines are equally important [8,9]. There is a paucity of knowledge on adapting generic guidelines in developing countries despite the huge demand [10,11]. This study aims to illustrate the process of adapting and scaling up a generic TB guideline to the China context and the experiences of those involved.

China uses a national policy guide [12] which contains information on TB control strategies, organisational requirements, case finding, TB chemotherapy and case follow-up. However, it lacks operational details demanded by the county level TB doctors. Routine in-service training sessions use the national policy guide, but the results are not satisfactory, partially due to the lack of operational details. Although good performance of the China DOTS programme has been reported in the last few decades [13,14], studies have also illustrated problems within the system including revenue-driven actions by the hospitals and TB dispensaries and loose TB case management [13,15-17]. The Nuffield Centre for International Health and Development at the University of Leeds developed a TB operational guideline (the *deskguide*) and an associated training module, which have been used to effectively scale up TB DOTS in all 124 districts in Pakistan. A generic version was drafted, field tested in Zambia and Cameroon, and published on the Nuffield website [18]. The deskguide contains detailed instructions on "how to do" TB daily case management. Though the deskguide cannot solve problems related to the wider health system, it was hoped a version adapted to the China context could improve TB case management in Chinese county TB dispensaries. We cooperated with the China National TB Programme (NTP) and two provincial TB programmes to adapt and scale up the deskguide for national use, with the aim of strengthening TB case management and improving the quality of TB care.

## Methods

### Settings

The adaptation was initiated in two provinces in agreement with the China NTP. Guangxi and Shandong provinces were selected to reflect the heterogeneity of China. Guangxi is located in the southwest of China bordering Vietnam. It is relatively poor with an average GDP per capita of $US 1,347 in 2006 (the national average is $US 2,010). Its landscape is hilly and mountainous, with relatively limited road accessibility. Minorities account for more than 40% of the population in Guangxi. In contrast, Shandong is on the east coast of China. It is relatively well-developed with a GDP per capita of $US 3500 in 2006. Shandong is largely flat with a few low hills, and has good roads. Over 95% of the population in Shandong are Han, the majority ethnic group in China. Guangxi and Shandong provinces are representative of China's low income western areas and well-developed eastern areas respectively.

### Contents of the deskguide and its training module

The deskguide is an operational guideline covering the major stages of TB case management: diagnosis of TB and prescribing drugs, preparing TB treatment, patient follow-up during treatment following the internationally recommended DOTS strategy for TB control, and assessment of treatment outcomes. Role-specific responsibilities of county TB clinicians and public health doctors are given for all tasks in detail in clear short sentences. The associated training module was designed for the needs of one-day in-service training workshops for county TB doctors (Table 1).

**Table 1:** Major content of the deskguide and its training module

The deskguide (operational guideline):
1) Diagnosis: identifying TB suspects, differentiating TB from similar diseases, TB diagnosis and classification, choosing treatment regimens and dosage;
2) Preparing TB treatment: educating patients, registering and arranging treatment, arranging and educating treatment supporters, screening household contacts;
3) Following up: visiting patients, reviewing patients, arranging sputum tests and changing treatment if necessary, retrieving lost patients, and managing patient interruptions;
4) Determining treatment outcomes: decision on treatment outcomes and ensuring quality of TB management.

The in-service training module included:
1) Introduction of the deskguide and how to use a guideline in practice;
2) Strengthening communication between doctors and TB patients;
3) Educating patients and choosing a treatment supporter;
4) Educating the TB supporter; and
5) Reviewing patients at the county TB dispensary.

The TB case management deskguide and training module (in Chinese and English) are free for public use by request to the corresponding author or visiting the following website:

InterventionThe deskguide was revised according to the national policy guide and adapted for practice in one prefecture of each province. In these prefectures, the deskguide and its training module were employed in the routine in-service training workshops for county TB staff, i.e., a one-day workshop twice a year (July 2005 to June 2006). Follow-up visits were conducted within two weeks of initial training to check for and address problems in using the deskguide. Included in the deskguide was the option of patients selecting a family member as their treatment supporter and guidance on how to train them in this role. Previously, according to the national policy guide only village doctors could be patient supporters, with the primary role of observing the patient to ensure they took each dose of their treatment.

In each province a control prefecture was selected. The pilot and control prefectures were selected to have similar population and economic development, similar TB notification rates and reported cure rates, and similar road accessibility (Table 2). In the control prefectures, the same numbers of in-service training workshops were given to county TB staff and similar numbers of supervisory trips were conducted. However, only the China national policy guide was used. The choice of family members was not available and village doctors were requested to observe the patient taking drugs.

**Table 2:** General information and TB control statistics from the intervention and control sites in Guangxi and Shandong, China

	Guangxi Province		Shandong Province	
	
	Pilot prefecture	Control prefecture	Pilot prefecture	Control prefecture
Population (2004)	2,450,000	2,297,000	5,500,000	5,640,000
Annual disposal income per head (2004) (RMB)*	2113	2122	3792	3334
Geography	Hilly	Hilly	Plain	Plain
Number of NSS+ patients registered in Jul–Dec 2004	412	362	700	758
Reported cure rates (%) for NSS+ patients registered in Jul–Dec 2004	91.0	93.1	97.4	96.9

* $1 US = 7.5 RMB in 2005

+NSS: new sputum smear positive TB cases

### Qualitative research methods

In March 2006, in-depth interviews and focus groups were conducted in the pilot and control prefectures to assess the usability of the deskguide and to compare the field experiences of using the deskguide with that of using the national policy guide only. In each prefecture, two focus groups of TB patients and two focus groups of family members were conducted with 5–9 participants per group (Table 3). TB patients who had completed more than two months of treatment were selected from the TB registers available at the county TB dispensaries. Their family member treatment supporters participated in a separate focus group interview. Questions asked in the focus groups included patient experience of communications with doctors on TB education, treatment support, drug renewals and side effects. Participants were chosen to ensure at least 25% of patients and family members were female.

**Table 3:** Number of interviewees in Guangxi and Shandong provinces in the qualitative study

	Guangxi Province		Shandong Province		Sub total
		
	Pilot prefecture	Control prefecture	Pilot prefecture	Control prefecture	
**Interviews**					

County TB doctors	4	4	5	3	16
Township public health doctors	4	4	4	4	16
Village doctors	4	5	4	4	17

**Focus group discussions**					

TB patients (2 focus groups in each prefecture)	14	15	18	16	63
Family members (2 focus groups in each prefecture)	12	11	18	16	57
Sub total	38	39	49	43	169

Four county TB doctors, four township public health doctors and four village doctors were interviewed using semi-structured interviews to explore their experience of treating and managing TB patients. Questions asked included "how often did you visit a patient?", "what did you say to the patient when you visited him/her", "what difficulties did you face in daily work and where to seek guidance in difficult cases?". Purposive sampling was used to select TB doctors from one relatively rich county and one relatively poor county of the prefecture. County, township and village TB doctors were selected from the same county as the TB patients to keep information consistent. Interviews and focus groups were conducted by members of the provincial staff.

Approval was obtained from the ethic committees of the Shandong and Guangxi provincial TB programmes. All TB patients, their family members and doctors participating in the research were asked for written informed consent: none refused.

### Data analysis

During all interviews and focus groups, notes were taken and audio recordings made, which were then transcribed onto computer. Thematic content analysis was employed to analyse the transcriptions. Transcripts were first categorised based on interview questions relevant to certain topics, and then three reviewers independently read the categorised transcripts and developed codes. All codes were discussed and merged into three key themes based on their recurrence and wide relevance: 1) the usability of the deskguide, 2) patient knowledge of TB and 3) patient experience of treatment support. Experience of TB case management was solicited both from the doctors and patients; however, patient reports were considered to be more reliable. Results of themes 2 and 3 were compared between the pilot and control sites to reflect the difference between using the deskguide and national policy guide together and using only the national policy guide.

## Process of adapting and scaling up the deskguide

### Eight steps of adaptation in two provinces

#### 1. Establish a steering committee and working groups

In May 2005, a steering committee was set up including the directors of China national and provincial TB programmes. This enabled the adaptation process to work effectively and the results be rapidly applied into practice. A small but efficient working group was formed in each province led by the provincial TB programme director. Each group had a focal person from the provincial TB programme who was familiar with procedures of TB case management and training. The focal person was the key to keeping effective communication among the members and ensuring an efficient working process of revising the deskguide.

#### 2. Review and revise the deskguide in accordance with national policy, local practice and training needs

The deskguide and its training module were translated into Chinese. Each chapter was reviewed and revised to be compatible with the national policy guide. Two major differences emerged. The first was related to China's TB organisation. The generic version of the guideline assumes that TB is managed under the district hospital as diagnostic centre with some health centres as treatment centres. This was revised for the China context where TB diagnosis and treatment are all managed by a county TB dispensary. Roles of the clinical and public health doctors of the county TB dispensary were defined according to their current practice. The second difference is TB treatment regimens and diagnosis. Intermittent instead of daily regimens are used in China, while both sputum smears and X-ray examination are requested for diagnosis according to the national policy guide.

The deskguide was also different from the national policy guide regarding who should provide direct observation of treatment (DOT) to TB patients. The national policy guide requested the village doctor to do DOT. However, in reality, most patients self-administer their treatment [17,19,20]. Evidence from several randomised controlled trials showed that DOT provided by community workers and family members can give the same or better treatment success rate as DOT by clinicians [21-24]. The WHO Stop-TB Partnership now recommends "supervision and patient support" in its new TB strategy [25]. The working group persuaded TB policy makers to allow a family member to be one of the options for patient treatment supporter: this change was reflected in the deskguide.

The training module was also revised. The generic module was designed for the training of doctors with no TB experience. However, in China TB doctors have already been trained, so a decision was made to incorporate the deskguide in the routine one-day in-service training. The module was then substantially reduced to focus on weak areas identified by the TB programmes: patient-doctor communication, patient education, selecting and educating treatment supporters and patient supervision.

#### 3. Hold workshops with practitioners at local levels

Participants at the county levels reviewed the deskguide and training module page by page, commenting on the appropriateness of phraseology and role-specific responsibilities based on their field experience.

#### 4. Hold a steering committee meeting with the NTP

During the first steering committee meeting in July 2005, the national TB directors approved the adaptation process and gave constructive comments regarding how to incorporate the deskguide training into routine in-service training workshops.

#### 5. Pilot the deskguide with immediate supportive supervision trips

In July 2005, all county TB doctors in the pilot prefectures were trained on the deskguide. Participatory learning approaches were used rather than the didactic lectures traditionally used in China. Trainees played the roles of the TB patient, doctor, and family member to practice how to select a TB treatment supporter and other communication skills. Initially TB doctors felt embarrassed to be involved in role-play; however, after encouragement and observing others' role-plays, they soon started to enjoy the process. At the end of the workshop, trainees reported that the role-plays had given them more vivid understanding of communication skills. The total numbers of county doctors trained in Guangxi and Shandong respectively were 23 (average age 34 years, 43% females) and 38 (average age 36 years, 24% females).

Supervisory trips to the county TB dispensaries were conducted within two weeks of the workshops to identify any problems arising at an early stage. We found that most TB doctors simply put the deskguide and training module in their bookshelves and continued their usual approach to case management. A second training workshop was quickly organised focusing on how to use the guideline in daily practice, i.e., putting it on the desk, familiarising its contents, and referring to it for guidance on unclear procedures.

#### 6. Revise the deskguide to reflect experience from practice

Revising, practising, reviewing and revising again substantially contributed to the precision of the final version of the deskguide. Checking on the use of the deskguide was incorporated into the routine supervisory trips to county TB dispensaries. During this stage, modest revisions were made on re-arranging the sequence of chapters and refining the detailed responsibilities of doctors.

#### 7. Conduct embedded operational research

to compare the practitioners' and patients' experiences of TB case management in the pilot prefectures with those in the control ones. The results facilitated the final acceptance of the deskguide for national use.

#### 8. Hold steering committee meetings to report progress and agree on changes

At these meetings, policy related issues were discussed, such as how the deskguide and training module fit into nationwide needs and in-service training for county TB doctors. Changes were made on operation issues: these were agreed with the NTP.

### National scale-up

The NTP approved the deskguide for national use in April 2006. Then a course on the deskguide and training module was provided at a national workshop with good feedback from the participants. The NTP published the deskguide and training module and disseminated the booklet to all provinces in Nov 2006 [26]. In December 2006, scale up of the deskguide began with Guangxi covering nearly 600 county TB doctors, led and financially supported by the Guangxi Provincial Health Bureau. Training of trainers was conducted for prefecture TB doctors; then the trainers trained county TB doctors in their own prefectures. A series of training documents were designed to ensure the quality of training, including the facilitator's guide, facilitator's module, trainee's module, the feedback sheet and supervision list (Table 4).

**Table 4:** Documents designed for the deskguide scale-up

**Document Names**	**Target**	**Major Contents**
The deskguide	Trainers and trainees	The major training document for the workshop. To be used on a daily basis.
The facilitator guide	Trainers	Facilitators' skills and procedures of the training workshop; Key points to be a good facilitator; Suggested training timetable for the day.
The facilitator modules	Trainers	Major contents of the deskguide to be taught; Actions of trainers needed in the workshop, such as letting trainees read, raising questions, leading discussions and role-plays.
The trainee modules	Trainees	Information for reading, discussion and role-plays with specific linkage with the deskguide.
The feedback sheet	Trainees	For trainees' feedback regarding their trainers and the workshop.
The supervision lists	Trainers	Specific areas to be checked using the deskguide during a supervisory trip to the county CDC. This will be done by the prefecture trainers within a month of the training and faxed to the provincial TB programme.

* All the finalised documents are in Chinese, with pre-final drafts also in English. All documents are free for public use by request to the corresponding author.

During review of the China TB national policy guide, the deskguide was incorporated as an essential component for case management at county levels. New national initiatives were added, namely the use of fixed dose combination drugs, the new diagnosis guidelines for sputum smear negative patients, and inter-provincial management of case transfers. The new version of the deskguide will be disseminated by the NTP as a complementary guideline with the new national policy guide in mid 2008 and its training will be incorporated into the new national policy guideline training workshops. These will train about 110,000 frontline county TB doctors nationwide in China.

## Results

The average age and sex distribution of the interviewees was similar between the pilot and control sites (Table 5). We interviewed more male doctors than females because there is a general gender imbalance in the public health work force. Pseudonyms are used in the quotes in this section to protect the confidentiality of respondents.

**Table 5:** Basic demographics of the interviewees in the pilot and control sites

		Pilot site interviewees	Control site interviewees	Total
County TB doctors	Average age (yrs)	40.5	38.3	39.6
	Number	9	7	16
	Male (%)	7 (77.8%)	6 (85.7%)	13 (81.3%)
Township public health doctors	Average age (yrs)	36.1	31.9	34.0
	Number	6 (75%)	6 (75%)	12 (75%)
	Male (%)	8	8	16
Village doctors	Average age (yrs)	38.2	44.8	41.7
	Number	8	9	17
	Male (%)	7 (87.5%)	7 (77.8%)	14 (82.4%)
TB patients	Average age (yrs)	50.3	50.0	50.1
	Number	32	31	63
	Male (%)	23 (71.9%)	18 (58.1%)	41 (65.1%)
Family members of TB patients	Average age (yrs)	44.9	41.2	43.1
	Number	30	27	57
	Male (%)	14 (46.7%)	16 (59.3%)	30 (52.6%)

### The deskguide was useful in the daily practice of county TB doctors

County TB doctors in the pilot sites reported that the deskguide was useful in solving problems faced in their daily practice. As Dr. L said, *"We did not have this stuff before. It [the deskguide] has detailed actions on diagnosis, patient education and training of the patient supporters"*. Dr. J reported, *"The deskguide is very comprehensive and practical. It is targeted for our local work"*; and Dr. W said,"*The deskguide has very useful details on diagnosis, patient education and treatment supporter training*." For instance, TB doctors used the information from the deskguide for patient education. Dr. X said, *"We always ask the patient to repeat the key points listed in the deskguide after the education. It helps them remember the knowledge"*. TB doctors copied the pages of patient education and treatment supporter education from the deskguide, and gave them to patients and their supporters after the consultation. Provincial TB programme staff reported from their supervision experience that most county TB doctors in the pilot prefectures put the deskguide on their desks and had personal reading marks on the pages. When asked about key components of the deskguide, e.g. how to select a treatment supporter, county TB doctors gave good answers in line with the deskguide requirements.

The major barrier to using the deskguide was reported as "*not being familiar with using a guideline in practice*": as Dr. Q continued, "*I simply follow the way we used to. If something is not clear, I ask colleagues rather than the deskguide*. " Understanding this, the working group re-emphasised how to use the guideline in the in-service training workshops and supervision trips. Also, pages regarding the TB diagnosis flowchart and key points on patient education were enlarged and put on the walls of doctors' offices. Comments for modest improvements such as *"The font can be bigger for better readability"*, and *"more information is needed on how to deal with side-effects" *were taken into consideration during the revision process.

### Patients in the pilot sites reported a better knowledge of the cause of TB and the importance of non-stopping treatment

As Patient T reported, "*The [county] doctor discussed with me for about 20 minutes when I was first diagnosed of TB. She told me that TB is infectious but can be cured. I need six months of treatment. She also answered my worries. I know from her that TB treatment is free". Patient M commented, "The doctor discussed with me for 15 minutes, and gave me a copy of the information. It is good as it helps my memory"*.

In the control sites, patients reported that county TB doctors told them that TB was contagious through coughing and spitting. However, many patients still had anecdotal ideas about TB. For example, Patient W said *"My TB was caused by eating unhealthy food. The county TB doctor dispensary did not know either." *Nearly half of the patients interviewed in the control sites could not answer the question regarding treatment duration of TB while all TB patients in the pilot prefectures knew it was six to eight months. In contrast to the pilot sites, many patients in the control sites reported the county TB doctor was busy and only spent a few minutes with them on their first consultation. As Patient S reported, *"the doctor told me that TB can be treated, but did not say how long [the treatment is]. He spent five minutes with me. He only told me come back when my drugs were finished"*.

### Better treatment support was reported in the pilot sites

In the pilot sites, county TB doctors helped patients select their treatment supporters, most of whom were family members. As one patient said, *"The doctor in the county TB dispensary was nice to me. He helped me find a person at home [her husband] to take care of my drugs"*. Patient Y said, *"My wife watched me taking drugs every time, then she marked the card. She also reminded me about the time for sputum tests"*. Most family members in the pilot group reported being trained on how to observe TB treatment, remind patients to take their drugs, and mark the treatment card. Some patients expressed concerns about stigma as a reason for not seeing the village doctor: as Patient H said, *"I felt very uneasy about catching TB. Young boys at my age in the village all go out [to the city] for work. I do not go. I do not want others to know my disease, even the neighbours and the village doctor. It is a good way that my Mom is watching me taking drugs"*.

In the control sites, family members reported they did help TB patients taking drugs occasionally, but not on a daily basis. As they had not been trained on treatment support, they did not have much knowledge of TB or TB treatment to support their relatives despite their strong desire to do so. Patients reported that they took the drugs by themselves except one who was watched by his brother as his brother was a village doctor. Patients reported various reasons why they had not been directly observed by the village doctor: "*I can take care of myself and do not need a stranger to watch me [taking drugs]"; "It takes 15 minutes to go to the village doctor's home and I am very busy in the morning"; and "I am afraid of being seen by others on the way to the doctor's home"*. Interviews with village doctors also revealed their unwillingness for treatment support, as they *"have no time", "do not feel the need to do so", and "the RMB 60 ($US 8) provided by the government for direct observation is too little"*.

In the pilot sites, all patients reported that they were given the telephone numbers of county TB doctors. More than one third of patients said they did call the doctor when they felt uneasy about taking drugs. County TB doctors were required to visit the problematic patients such as defaulters and patients with serious side-effects. There was no difference in the frequency of patient visits by county TB doctors or township doctors between the pilot and control sites, but a better record of patient visits was seen in the pilot sites. Nine out of 31 patients in the control sites reported that they had never been seen by a village doctor. Township doctor interviews also showed that village doctors did not watch patients taking drugs. For example, Dr. H said, *"In my township no village doctor actually supervises TB patients taking their drugs. They [village doctors] are more concerned about earning money. They can easily get much more from one treatment than the government fee for supervising a patient over six months"*.

More patients in the control sites reported missing doses of their drugs during treatment (8/31 reported missing in the control sites vs. 3/32 in the pilot sites). Common reasons given for drug omissions were side-effects such as feeling nauseous or vomiting. An interruption of two weeks was identified in the control site.

## Discussion

There are many reports of the challenges of getting research findings into practice [10,27], such as the lack of evidence-based pilots [28], the autonomy of medical professionals [29], policy-makers not using research evidence for decision-making [30], passive dissemination methods [10] and resource barriers. However, there are few publications reporting successful experience in this field. This case study attempts to redress this imbalance. A number of the lessons learnt in this project may guide the adaptation of other generic guidelines in developing countries.

First, giving local policy-makers and practitioners a lead in the process was crucial for making changes in policy and practice. The design and implementation of the deskguide were conducted in close partnership with local TB programmes: the directors of the China NTP were involved in the steering committee, and the provincial TB directors led their own working groups. This collaborative approach ensured ownership of the final products by the TB programmes. Each provincial working group repeatedly updated the deskguide to reflect changes identified in the pilot. Other national initiatives which the China NTP considered important were added during the scale-up, making the deskguide a true NTP product.

Second, the approach to adaptation and scale-up was systematic. Traditional approaches such as publishing research papers in peer reviewed journals and/or on websites, are relevant but not sufficient to bring change in guideline practice [31,32]. The eight-step working group process described here included reviewing, revising, piloting, and revising the materials again reflecting field experience. The key element for an effective working group was the focal person, who acted as a bridge between the policy makers and practitioners. It was very helpful that the focal persons came from the TB programme at a higher level. This meant that not only were they capable of revising the files in a timely manner according to comments from both the policy-makers and the frontline TB doctors; but also, that they were able to communicate in a timely fashion with county TB doctors through routine supervisory trips.

Third, the adapted guideline and other materials were replicable and sustainable for scale-up over a wider area. The deskguide and its training module were designed to fit the in-service training workshops which were routinely provided for TB doctors. The facilitators' guide provided specific information on the training methods and the timetable in a user-friendly way. This ensured that the quality of training was better maintained during the cascade training from national to county levels that is used in China and other large countries. The follow-up and periodical field supervision trips were important to support county TB doctors in using the deskguide, especially at the initial stage. All these activities and documents were designed to fit into the routine system, and were funded by the TB programmes, so that no additional funding was required after the initial pilot courses and training of trainers at the national and provincial levels.

Fourth, embedded operational research provided evidence for national acceptance. The qualitative research, embedded in the adaptation process, identified that county TB doctors regarded the deskguide as useful for their daily practice. Patients had better TB knowledge and better treatment support in the pilot sites, reflecting a better quality of TB care.

The case study reported here had three limitations. First, due to the nature of case studies, only one prefecture in each of two provinces could be chosen for the pilot, together with one other prefecture within each province, chosen for comparative purposes. Economic development and road accessibility were the two major factors considered because general economic development influenced the human resources and capacity of the TB control system, and these two factors both affect patient access to care and treatment adherence [15,19,33]. The China NTP considered that the deskguide would be appropriate for other parts of China if it was successful in the two contrasting provinces. The study was intended to illustrate the process of adapting the deskguide in China to help inform policy: it was not intended to provide quantitative data to test hypotheses about the effectiveness of the deskguide and training module. As anticipate, the adaptation process produced a useful package of the deskguide and other deliverables for the China TB programme. Second, provincial staff could have been biased when conducting the qualitative interviews and focus groups. It was not possible to employ outside researchers due to time limitations and the need for interviewers to have knowledge about TB case management. Also, the operational study could not be implemented in a blinded fashion. However, provincial TB staff were expected to have been less biased than prefecture or county level staff as they were not directly related to the pilot. All provincial TB staff who conducted interviews were given two days training on interview skills and neutrality. Third, costs of the deskguide adaptation were not collected. This is because the deskguide training was designed and incorporated into the routine in-service training which had already been budgeted and paid for by the TB programme. The overall cost of the process and research was only $US 46,000, a modest amount given its national influence in China.

## Conclusion

This case has shown how a generic TB operational guideline and its training module were adapted and scaled up in China. A number of lessons can be learned that are useful in more general adaptation processes: the importance of involving local policy makers and practitioners; the need to employ a systematic adaptation approach using capable focal persons; and the need to align guidelines with current strategies and practice so they will be sustainable and replicable for scale-up. The description of the process given here can provide insights for policy-makers and researchers on how to adapt and scale up other generic guidelines in developing countries.

## Abbreviations

TB: Tuberculosis; DOTS: the brand name of tuberculosis control programme recommended by the World Health Organisation; DOT: directly observed treatment; NTB: National TB Programme.

## Competing interests

The authors declare that they have no competing interests.

## Authors' contributions

XW and JDW were involved in drafting the paper while XL and XZ have provided critical comments. All authors were actively involved in designing adaptation process, implementing the deskguide and its training module, and conducting the embedded qualitative research. XW, XL, JDW and XZ were extensively involved in revising the adapted deskguide and training module. XL and FL led the implementation of deskguide in Guangxi while XZ and RL led the implementation of deskguide in Shandong. All authors read and approved the final manuscript.

## Pre-publication history

The pre-publication history for this paper can be accessed here:



## References

[B1] WHO, ATS, American Lung Association, KNCV, CDC, WHO (2003). Management of Tuberculosis: training for health facility staff.

[B2] Maher D, Chaulet P, Spinaci S, Harries A (1997). Treatment of Tuberculosis: Guidelines for National Programmes.

[B3] Woolf SH, Grol R, Hutchinson A, Eccles M, Grimshaw J (1999). Clinical guidelines: potential benefits, limitations, and harms of clinical guidelines.. BMJ.

[B4] Garner P, Meremikwu M, Volmink J, Xu Q, Smith H (2004). Putting evidence into practice: how middle and low income countries "get it together".. BMJ.

[B5] McMichael C, Waters E, Volmink J (2005). Evidence-based public health: what does it offer developing countries?. Journal of Public Health.

[B6] Walley J, Khan A, Karam S, Witter S, Wei X (2007). How to get research into practice - first get research into research: lessons learned from TB partnerships in Pakistan and China. Bulletin of the World Health Organization.

[B7] Aaserud M, Lewin S, Innvaer S, Paulsen EJ, Dahlgren AT, Trommald M, Duley L, Zwarenstein M, Oxman AD (2005). Translating research into policy and practice in developing countries: a case study of magnesium sulphate for pre-eclampsia. BMC Health Services Research.

[B8] Health Services Research Group (1992). Standards, guidelines and clinical policies. Health Services Research Group.[comment]. CMAJ.

[B9] Cluzeau FA, Littlejohns P, Grimshaw JM, Feder G, Moran SE (1999). Development and application of a generic methodology to assess the quality of clinical guidelines. International Journal for Quality in Health Care.

[B10] Bero L, Grilli R, Grimshaw M, Harvey E, Oxman A, Thomson M (1998). Getting research findings into practice: closing the gap between research and practice: an overview of systematic reviews of interventions to promote the implementation of research findings. BMJ.

[B11] De Muynck A, Siddiqi S, Ghaffar A, Sadiq H (2001). Tuberculosis control in Pakistan: critical analysis of its implementation. JPMA J Pak Med Assoc.

[B12] Disease Control Division MoH China (2002). China Tuberculosis Prevention and Control Plan: guideline for programme implementation.

[B13] Wang DL, Liu JJ, Chin D (2007). Progress in tuberculosis control and the evolving public-health system in China. Lancet.

[B14] Zhao FZ, Zhan Y, Liu XQ (2003). Tuberculosis control in China. Tuberculosis.

[B15] Tang S, Squire SB (2005). What lessons can be drawn from tuberculosis control in China in the 1990's? An analysis from a health system perspective. Health Policy.

[B16] Meng Q, Li R, Cheng G, Blas E (2004). Provision and financial burden of TB services in a financially decentralized system: a case study from Shandong, China. Int J Health Plann Manage.

[B17] Zhan S, Wang L, Yin A, Blas E (2004). Revenue-driven in TB control--three cases in China. Int J Health Plann Manage.

[B18] Nuffield Centre Generic guidelines for community based TB DOTS.

[B19] Disease Control Department, Ministry of Health (2006). General report of social evaluative studies on TB control programmes. China TB Control Program: Social evaluative study report 2004-2005.

[B20] Hu D, Liu X, Cheng J, Wang Y, Wang T, Zeng W, Smith H, Garner P (2008). Direct observation and adherence to tuberculosis treatment in Chongqing, China: a descriptive study. Health Policy and Planning.

[B21] Zwarenstein M, Schoeman JH, Vundule C, Lombard CJ, Tatley M (2000). A randomised controlled trial of lay health workers as direct observers for treatment of tuberculosis. Int J Tuberc Lung Dis.

[B22] Newell JN, Baral SC, Pande SB, Bam DS, Malla P (2006). Family-member DOTS and community DOTS for tuberculosis control in Nepal: cluster-randomised controlled trial.. Lancet.

[B23] Walley J, Khan A, Newell J, Khan MH (2001). Effectiveness of the direct observation component of DOTS for tuberculosis: a randomised controlled trial in Pakistan. Lancet.

[B24] Wright J, Walley J, Philip A, Pushpananthan S, Dlamini E, Newell J, Dlamini S (2004). Direct observation of treatment for tuberculosis: a randomized controlled trial of community health workers versus family members. Tropical Medicine & International Health.

[B25] Uplekar M (2006). The STOP TB Strategy: Building on and enhancing DOTS to meet the TB related Millennium Development Goals.

[B26] Wei X, Liang X, Walley J, Liu F, Zhang X, Li R (2006). China Tuberculosis Case Management Deskguide and the Training Module.

[B27] Haider M, Kreps GL (2004). Forty years of diffusion of innovations: utility and value in public health. Journal of Health Communication.

[B28] Buekens P, Keusch G, Belizan J, Bhutta Z (2004). Evidence-based global health. JAMA.

[B29] Dopson S, Locock L, Gabbay J, Ferlie E, Fitzgerald L (2003). Evidence based medicine and the implementation gap. Health: An interdisciplinary journal for the social study of health, illness and medicine.

[B30] Wei X (2004). Evaluation of the Effect of a Diabetes Management Program on Continuity of Care in Shanghai. Health Policy Management and Evaluation.

[B31] Davis DA, Thomson MA, Oxman AD, Haynes RB (1995). Changing physician performance. A systematic review of the effect of continuing medical education strategies.. JAMA.

[B32] Solberg LI, Brekke ML, Fazio CJ, Fowles J, Jacobsen DN, Kottke TE, Mosser G, O'Connor PJ, Ohnsorg KA, Rolnick SJ (2000). Lessons from experienced guideline implementers: attend to many factors and use multiple strategies. Joint Commission Journal on Quality Improvement.

[B33] Wang WB, Jiang QW, Chen Y, Xu B (2007). Pathways from first health care seeking to diagnosis: obstacles to tuberculosis care in rural China. Int J Tuberc Lung Dis.

